# 7α-Hydroxycholesterol induces monocyte/macrophage cell expression of interleukin-8 via C5a receptor

**DOI:** 10.1371/journal.pone.0173749

**Published:** 2017-03-21

**Authors:** Hyok-rae Cho, Yonghae Son, Sun-Mi Kim, Bo-Young Kim, Seong-Kug Eo, Young Chul Park, Koanhoi Kim

**Affiliations:** 1 Department of Neurosurgery, Kosin University, College of Medicine, Seo-gu, Busan, Republic of Korea; 2 Department of Pharmacology, Pusan National University—School of Medicine, Yangsan, Gyeongnam, Republic of Korea; 3 College of Veterinary Medicine and Bio-Safety Research Institute, Chonbuk National University, Iksan, Jeonbuk, Republic of Korea; 4 Department of Microbiology and Immunology, Pusan National University—School of Medicine, Yangsan, Gyeongnam, Republic of Korea; University of Hong Kong, HONG KONG

## Abstract

We investigated effects of 7-oxygenated cholesterol derivatives present in atherosclerotic lesions, 7α-hydroxycholesterol (7αOHChol), 7β-hydroxycholesterol (7βOHChol), and 7-ketocholesterol (7K), on IL-8 expression. Transcript levels of IL-8 and secretion of its corresponding gene product by monocytes/macrophages were enhanced by treatment with 7αOHChol and, to a lesser extent, 7K, but not by 7βOHChol. The 7-oxygenated cholesterol derivatives, however, did not change transcription of the IL-8 gene in vascular smooth muscle cells. 7αOHChol-induced IL-8 gene transcription was inhibited by cycloheximide and Akt1 downregulation, but not by OxPAPC. Expression of C5a receptor was upregulated after stimulation with 7αOHChol, but not with 7K and 7βOHChol, and a specific antagonist of C5a receptor inhibited 7αOHChol-induced IL-8 gene expression in a dose dependent manner. Pharmacological inhibitors of PI3K and MEK almost completely inhibited expression of both IL-8 and cell-surface C5a receptor induced by 7αOHChol. These results indicate that 7-oxygenated cholesterol derivatives have differential effects on monocyte/macrophage expression of IL-8 and C5a receptor and that C5a receptor is involved in 7αOHChol-induced IL-8 expression via PI3K and MEK.

## Introduction

Interleukin-8 (IL-8)/CXCL8, a member of the CXC chemokine family, forms the first line in host defense by activating and recruiting neutrophils to the site of injury or infection [1, 2]. IL-8 also induces firm adhesion of monocytes expressing CXCR2, a CXCL8 receptor, to the endothelium [1, 3]. Besides the physiological functions, clinical and animal studies indicate a role of IL-8 in the pathogenesis of atherosclerosis. Macrophage expression of IL-8 is significantly elevated in human atherosclerotic lesions [4, 5], and atherosclerosis is significantly reduced in animals deficient in IL-8 [3]. Therefore, identification of lipid molecules responsible for IL-8 elevation in atherosclerotic lesions will provide a better understanding of the early stages of atherogenesis.

Cholesterol is present in both intracellular and extracellular forms in human atherosclerotic lesions, and the extracellular forms undergo oxidative modification to cholesterol oxides (oxysterols) [6, 7]. Major oxysterols identified from atherosclerotic human aorta include either 27-hydroxycholesterol (27OHChol) or 7-oxygenated cholesterol derivatives, such as 7-ketocholesterol (7K), 7β-hydroxycholesterol (7βOHChol), and 7α-hydroxycholesterol (7αOHChol) [8, 9]. Oxygenated cholesterol molecules have been reported to modify CXCL8 production [10, 11]. However, it is unknown how the 7-oxygenated cholesterol derivatives affect macrophage expression of IL-8.

In order to identify extracellular cholesterol oxidation product involved in elevated levels of IL-8, we evaluated the effectiveness of the 7-oxygenated cholesterol molecules on IL-8 expression using the human monocyte/macrophage (THP-1) cells. We also attempted to determine cellular molecules involved in IL-8 expression in response to cholesterol oxidation product to understand molecular mechanisms underlying dysregulated elevation of the chemokine in atherosclerotic lesions.

## Materials and methods

### Cells and reagents

Human monocyte/macrophage (THP-1) cells were purchased from the American Type Culture Collection (Manassas, VA, USA). Human aortic smooth muscle cells (HAoSMCs) purchased from Cambrex (East Rutherford, NJ) were grown in Dulbecco’s modified Eagle’s medium-high glucose (DMEM) supplemented with 15% FBS, 50 units/ ml penicillin and 50 μg/ml streptomycin in a humidified atmosphere of 5% CO_2_. 7αOHChol and 7β OHChol were purchased from Research Plus, Inc. (Barnegat, NJ, USA). 7K and LY294002 were acquired from Sigma-Aldrich (St. Louis, MO, USA). U0126 was purchased from Calbiochem Cell Signaling (San Diego, CA, USA). W-54011 and anti-C5a receptor antibody were purchased from Santa Cruz Biotechnology (Santa Cruz, CA, USA). Lipopolysaccharide (LPS), OxPAPC, and polymyxin B were purchased from InvivoGen (San Diego, CA, USA).

### Reverse transcription (RT)-polymerase chain reaction (PCR)

After reverse-transcription of total RNAs for 1 h at 42°C with Moloney Murine Leukemia Virus reverse transcriptase, transcripts of the IL-8 gene were amplified by RT-PCR or assessed by real-time PCR using a LightCycler® 96 Real-Time PCR System (Roche, Germany) as described [12]. PCR was performed using *Taq* PCR Kit. The cDNA was denatured at 90°C for 5 min followed by 25 cycles of PCR (95°C for 30 sec, 55°C for 30 sec, 72°C for 30 sec). The RT-PCR primers were IL-8: 5’-gtgcagttttgccaaggagt-3’ (forward) and 5’-acttctccacaaccctctgc-3’ (reverse); C5a receptor: 5’-gccttggtcatctttgcagt-3’ (forward) and 5’-caggaaggagggtatggtca-3’ (reverse); GAPDH: 5’-gagtcaacggatttggtcct-3’ (forward) and 5’-tgtggtcatgagtccttcca-3’ (reverse). Real-time quantitative PCR was performed in triplicate using the LightCycler 96 Real-Time PCR System (Roche, Germany); each 20-μl reaction consisted of 10 μl of SYBR Green Master Mix, 2 μl of forward and reverse primers (10 pM each) of genes to be analyzed, and cDNA template. Thermal cycling conditions were as follows: 95°C for 10 min, and 45 cycles at 95°C for 10 sec, 50°C for 10 sec, and an elongation period for 10 sec at 72°C. The relative expression of each gene was then calculated as a ratio to GAPDH using LightCycler 96 software (Version 1.1.0.1320). The real-time PCR primers were IL-8: 5’-actgagagtgattgagagtggac-3’ (forward) and 5’-aaccctctgcacccagttttc-3’; GAPDH: 5’-atggggaaggtgaaggtcg-3’ (forward) and 5’-ggggtcattgatggcaacaata-3’ (reverse).

### Enzyme-linked immunosorbent assay (ELISA)

The levels of CXCXL8 secreted into the culture medium were measured using a commercially available enzyme-linked immunosorbent assay (ELISA) kit (BD OptEIA^TM^ Human IL-8 ELISA Kit II) according to the manufacturer’s instructions (BD Biosciences, San Diego, CA, USA).

### Flow cytometric analysis

After incubation with 7-oxygenated cholesterol derivatives, THP-1 cells were harvested and incubated for 1 h at 4°C with antibody against C5a receptor, followed by multiple washings and incubation with fluorescent dye-conjugated secondary antibody. Cells were washed and resuspended in 1% paraformaldehyde in phosphate buffered saline. Flow cytometry was performed for analysis of fluorescence.

### Statistical analysis

Statistical analyses were performed using one-way ANOVA, followed by Dunnett’s multiple comparison test with the GraphPad PRISM software (GraphPad Software Inc., San Diego, CA). A P-value less than 0.05 was considered to be statistically significant.

## Results

### The features of IL-8 transcription induced by 7αOHChol

**Fig 1:**
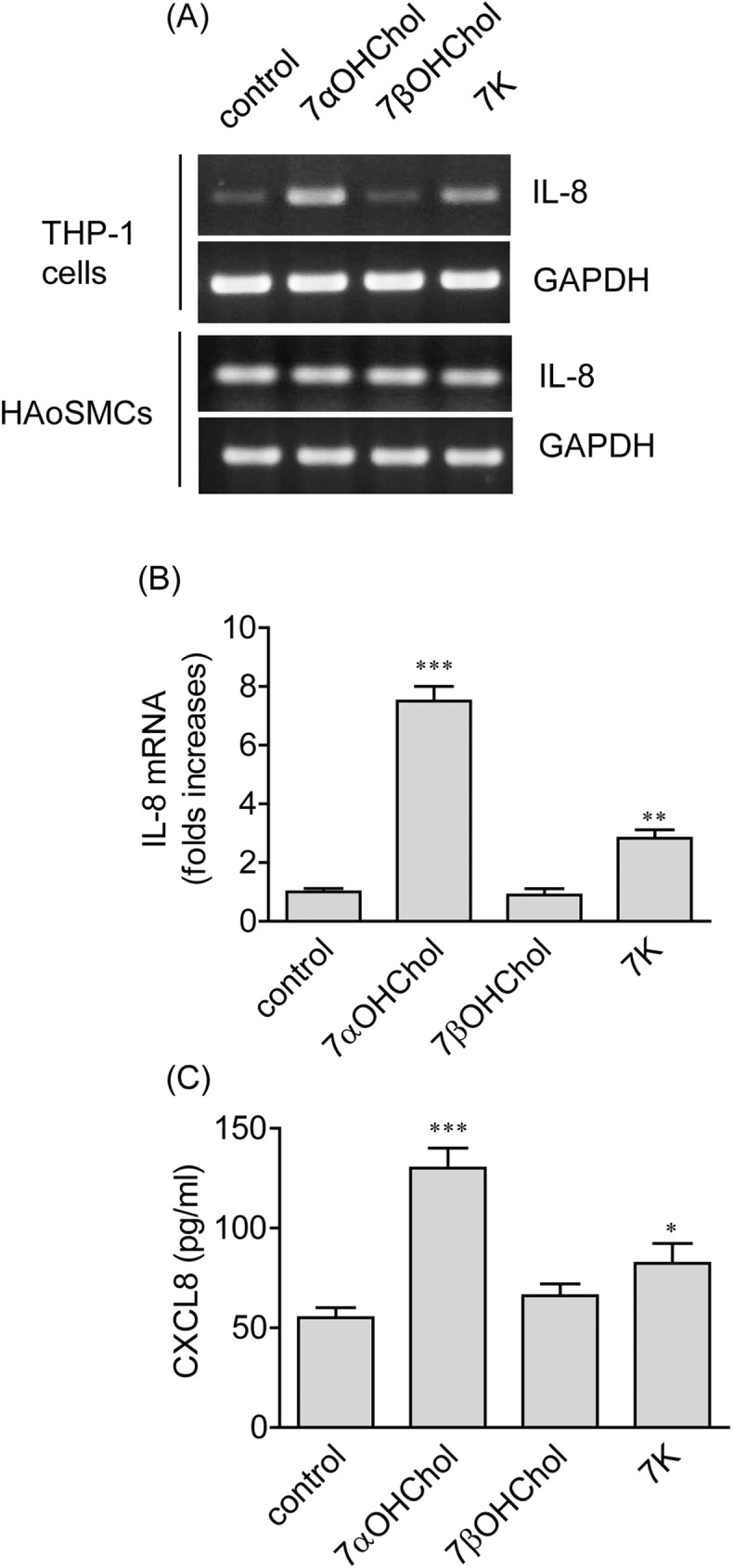
Effects of 7-oxygenated cholesterol derivatives on expression of the IL-8 gene. (A) THP-1 cells and human aortic smooth muscle cells (HAoSMCs) were incubated with or without the indicated 7-oxygenated cholesterol derivatives (5 μg/ml each) for 48 h. Transcripts of IL-8 were amplified by RT-PCR. (B) THP-1 cells serum-starved for 24 h in RPMI 1640 containing 0.1% BSA (endotoxin free) were incubated with or without 7-oxygenated cholesterol derivatives for 48 h, and the levels of IL-8 transcripts were assessed by real-time PCR. The y-axis values represent fold increases of IL-8 mRNA levels normalized to GAPDH levels relative to those of THP-1 cells incubated carrier of oxygenated cholesterol derivatives (control). Data are expressed as the means ± SD (n = 3 replicates for each group). **P < 0.01 vs. control; ***P < 0.001 vs. control. (C) Conditioned media were harvested after treatment of THP-1 cells (2 x10^5^ cells/ml) with or without 7-oxygenated cholesterol derivatives (5 μg/ml each) for 48 h. The amount of CXCL8 secreted into the media was measured by ELISA. Data are expressed as the means ± SD (n = 3 replicates for each group). *P < 0.05 vs. control; *** P < 0.001 vs. control.

We investigated whether 7-oxygenated cholesterol derivatives such as 7αOHChol, 7βOHChol, and 7K affected expression of the IL-8 gene of vascular smooth muscle cells and THP-1 cells. The 7-oxygenated cholesterol derivatives did not enhance IL-8 expression in human aortic smooth muscle cells. IL-8 transcripts were faintly detected from THP-1 cells, and the transcripts were increased after stimulation with 7αOHChol or 7K (Fig 1A). Levels of IL-8 transcripts were elevated by 7.5- and 2.8-folds after treatment with 7αOHChol and 7K, respectively, as determined by quantitative realtime PCR. In contrast, 7βOHChol did not affect the levels of IL-8 gene transcripts (Fig 1B). We determined whether these cholesterol derivatives affected production of the IL-8 gene product. The amount of CXCL8 secreted from THP-1 cells was significantly elevated upon stimulation with 7αOHChol and, to a lesser extent, 7K whereas 7βOHChol did not enhance secretion of CXCL8 (Fig 1C). The lack of activity of 7βOHChol was not due to cytotoxicity because it did not reduce viability of THP-1 cells (S1 Fig). Collectively, these results mean that, among 7-oxygenated cholesterol derivatives, 7αOHChol efficiently enhances expression of IL-8 in monocytes/macrophages.

**Fig 2:**
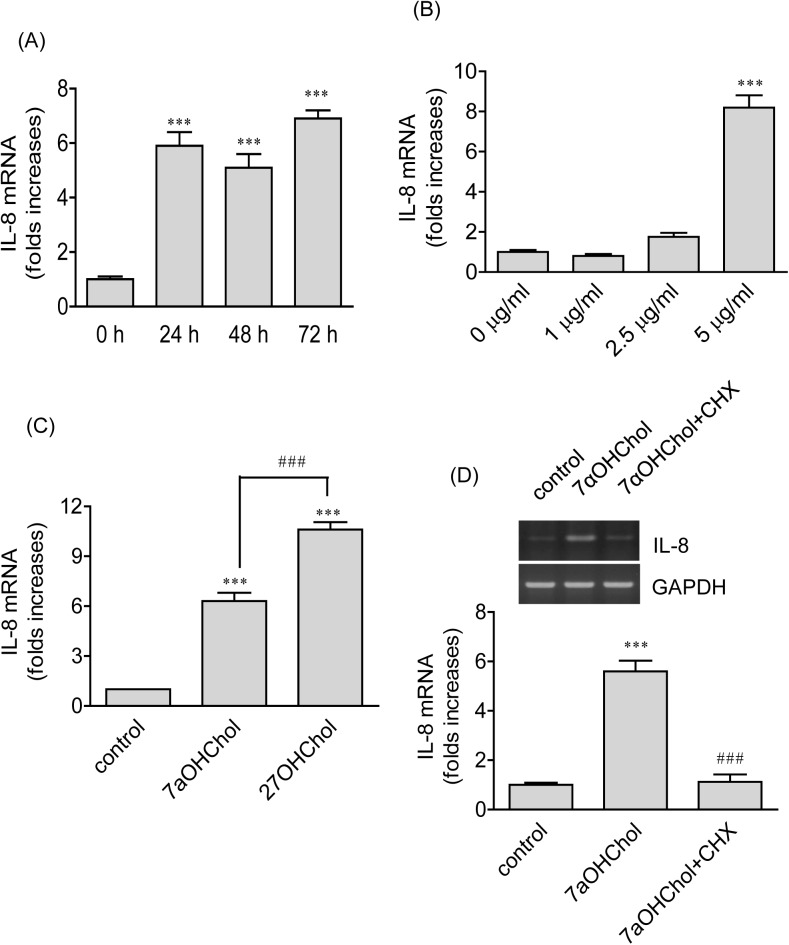
Effects of 7αOHChol on expression of IL-8. (A) THP-1 cells were incubated with 7αOHChol (5 μg/ml) for the indicated time periods. The levels of IL-8 transcripts were assessed by real-time PCR. Data are expressed as the means ± SD (n = 3 replicates for each group). * P < 0.05 vs. 0 h; *** P < 0.001 vs. 0 h. (B) THP-1 cells were incubated in the presence of the indicated amounts of 7αOHChol for 48 h. The levels of IL-8 transcripts were assessed by real-time PCR. Data are expressed as the means ± SD (n = 3 replicates for each group). *** P < 0.001 vs. 0 μg/ml. (C) THP-1 cells were treated for 48 h with 7αOHChol (5 μg/ml) and 27OHChol (2.5 μg/ml). The levels of IL-8 transcripts were assessed by realtime-PCR. Data are expressed as the means ± SD (n = 3 replicates for each group). *** P < 0.001 vs. control. (D) THP-1 cells were incubated for 48 h with or without 7αOHChol (5 μg/ml) in the absence or presence of CHX (1 μg/ml). Transcripts of IL-8 were amplified by RT-PCR (upper panel), and the levels of IL-8 transcripts were determined by real-time PCR (lower panel). Data are expressed as the means ± SD (n = 3 replicates for each group). *** P < 0.001 vs. control; ### P < 0.001 vs. 7αOHChol.

We conducted time course and concentration experiments using 7αOHChol. Significantly enhanced gene transcription of IL-8 occurred at 24 h post-treatment with 7αOHChol and maintained for up to 72 h after treatment (Fig 2A). Gene transcription of IL-8 was significantly elevated in the presence of 5 μg/ml of 7αOHChol, but not in the presence of small amounts such as 1 or 2.5 μg/ml of 7αOHChol (Fig 2B). We assessed the effectiveness of 7αOHChol on IL-8 expression in comparison with that of 27OHChol. The two oxidized cholesterol molecules elevated transcription of the IL-8 gene, and of the two molecules 27OHChol more efficiently elevated transcript levels of IL-8 (Fig 2C).

We determined whether transcription of the IL-8 gene induced by 7αOHChol necessitated protein synthesis using cycloheximide (CHX) which exerts its effect by interfering with the translocation step in protein synthesis. Treatment with CHX resulted in complete inhibition of 7αOHChol-induced IL-8 gene transcription (Fig 2D), suggesting necessity of de novo protein synthesis for 7αOHChol to elevate IL-8 transcripts. We investigated whether Toll-like receptor (TLR)-2/4 was involved in 7αOHChol-induced IL-8 expression using OxPAPC, a TLR2 and TLR4 inhibitor. The IL-8 gene transcription induced by peptidoglycan was profoundly reduced in the presence of OxPAPC whereas OxPAPC did not reduce transcript levels of IL-8 enhanced by 7αOHChol, suggesting a role of TLR-2/4 in IL-8 expression induced by peptidoglycan, but not by 7αOHChol (S2 Fig). Endotoxins that contaminate laboratory reagents and cell preparations can play roles in chemokine expression. To rule out this possibility, we used polymyxin B, an agent that binds to LPS and prevents its biological effects. Transcription of the IL-8 gene induced by LPS was almost completely blocked in the presence of polymyxin B whereas polymyxin B did not affect transcript levels of IL-8 increased by 7αOHChol (S3 Fig).

### Roles of the phosphoinositide-3-kinase (PI3K)/Akt1 and the mitogen-activated protein/extracellular signal-regulated kinase kinase (MEK) in 7αOHChol-induced expression of IL-8

**Fig 3:**
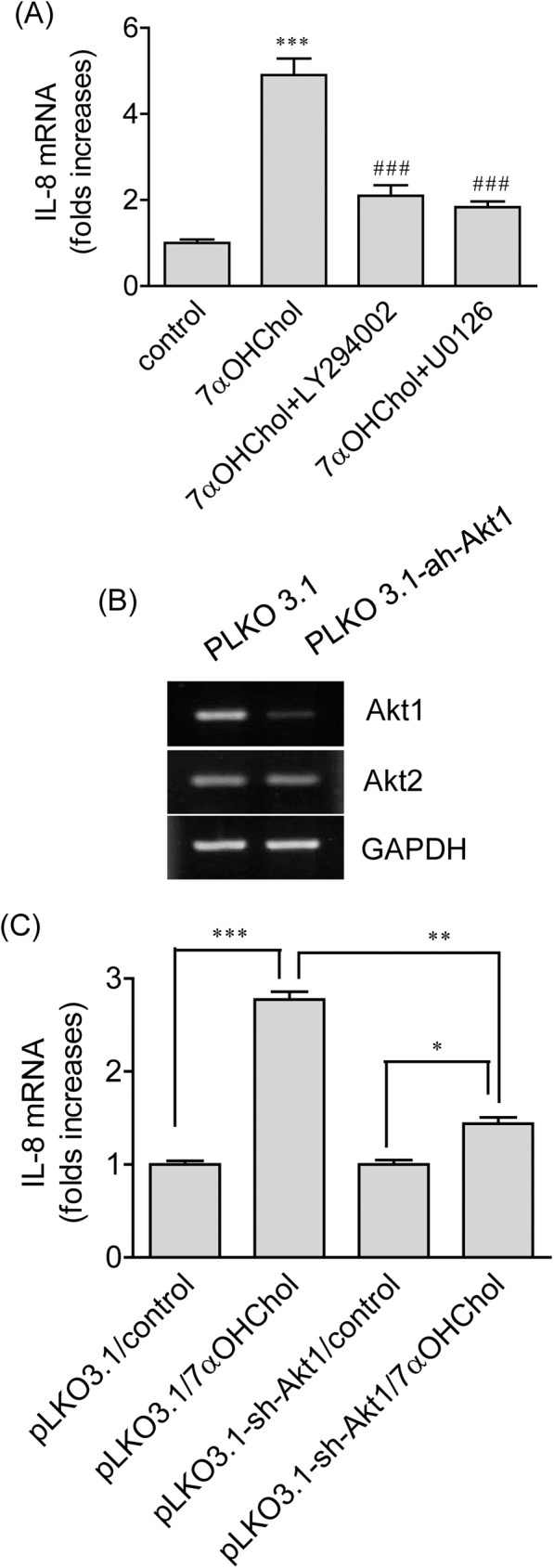
Effects of inhibition of PI3K/Akt1 and MEK on 7αOHChol-induced IL-8 expression. (A) THP-1 cells were treated with 7αOHChol for 48 h in the absence or presence of LY294002 or U0126 (10 μM each). Transcript levels of IL-8 were assessed by realtime-PCR. Data are expressed as the means ± SD (n = 3 replicates for each group). *** P < 0.001 vs. control; ## P < 0.01 vs. 7αOHChol; ### P < 0.001 vs. 7αOHChol. (B) THP-1 cells were infected with lentiviruses expressing Akt1 shRNA or control lentiviruses. After selection in the presence of puromycin, expression of Akt1 transcripts was examined by RT-PCR. (C) THP-1 cells infected with lentiviruses expressing Akt1 shRNA or control lentiviruses were stimulated for 48 h with or without 7αOHChol. Transcript levels of IL-8 were assessed by realtime-PCR. Data are expressed as mean ± SD (n = 3 replicates/group). * P < 0.05; ** P < 0.01; *** P < 0.001.

Treatment of THP-1 cells with 7αOHChol causes phosphorylation of Akt and ERK1/2 [13]. Therefore, we examined whether the PI3K and MEK were involved in 7αOHChol-induced expression of IL-8 (Fig 3A). Inhibition of PI3K using LY294002 resulted in significantly attenuated transcription of the IL-8 gene, and treatment with U0126, which inhibits activation of ERK1/2, led to profoundly decreased levels of IL-8 transcripts. We attempted to investigate the role of Akt1 isoform in IL-8 expression. Akt1-knockdown cells were generated by infecting lentiviruses encoding *Akt1* shRNA. When knockdown efficiency was determined by RT-PCR, profoundly reduced transcript levels of Akt1 were observed from cells infected with lentiviruses encoding *Akt1* shRNA, but not in cells infected with control lentiviral vector (Fig 3B). Treatment of THP-1 cells infected with control lentiviral vector with 7αOHChol caused enhanced transcription of the IL-8 gene. However, the enhanced transcription by 7αOHChol was impaired in Akt1-knockdown cells (Fig 3C). Collectively, these results strongly suggest that PI3K, MEK, and Akt1 are involved in 7αOHChol-induced IL-8 expression.

### Involvement of C5a receptor in 7αOHChol-induced CXCL8 production

**Fig 4:**
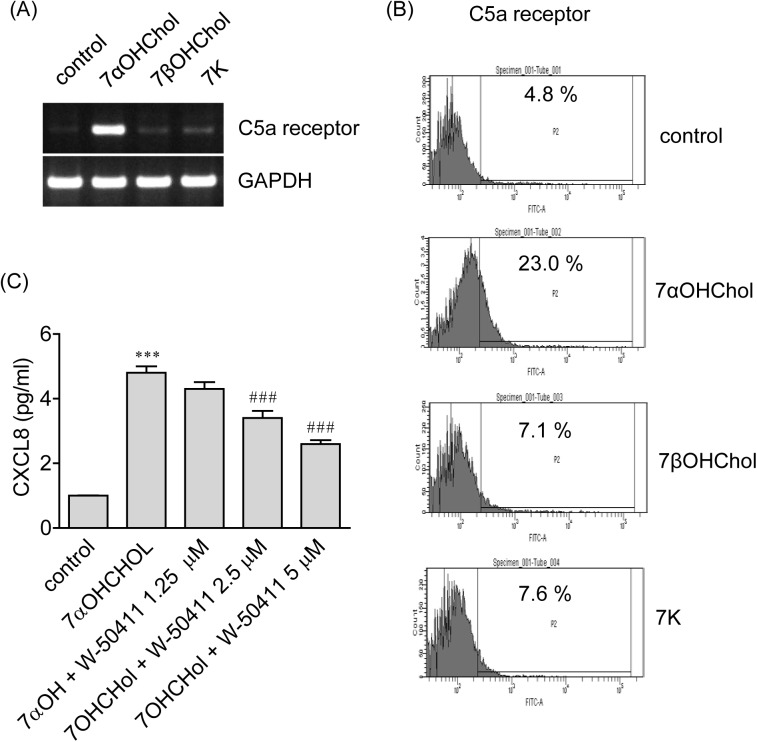
Roles of C5a receptor in on 7αOHChol-induced IL-8 expression. (A) THP-1 cells were incubated with or without 7-oxygenated cholesterol derivatives (5 μg/ml each) for 48 h. Transcripts of C5a receptor were amplified by RT-PCR. (B) THP-1 cells were incubated with or without 7-oxygenated cholesterol derivatives for 48 h, and surface expression of C5a receptor was analyzed by flow cytometry. (C) THP-1 cells were pre-treated for 2 h with the indicated amount of W-54011 and stimulated with 7αOHChol (5 μg/ml) for 48 h. Transcript levels of IL-8 were assessed by real-time PCR. Data are expressed as mean±SD (n = 3 replicates for each group). ***P < 0.001 vs. control. ### P < 0.001 vs. 7αOHChol.

Because C5a receptor modulates production of the IL-8 gene product from human cells [14], we investigated involvement of the receptor in 7αOHChol-induced IL-8 expression. First, we determined effects 7-oxygenated cholesterol derivatives on expression of C5a receptor. Transcripts of C5a receptor were faintly detected from THP-1 cells, and the transcripts were elevated after treatment with 7αOHChol. The elevation, however, was not observed after treatment with 7K or 7βOHChol (Fig 4A). The surface expression of C5a receptor was elevated mainly by treatment with 7αOHChol. The percentage of unstimulated control positive for C5a receptor was 4.8%, and it increased to 23.0% by treatment with 7αOHChol (Fig 4B). We investigated involvement of C5a receptor in IL-8 expression using W-54011, a specific C5a receptor antagonist. 7αOHChol elevated transcript levels of IL-8, but the elevation was significantly reduced by treatment with W-54011 in a dose-dependent manner (Fig 4C). These results mean that 7αOHChol upregulates C5a receptor which mediates 7αOHChol-induced IL-8 expression.

### Roles of the PI3K and the MEK in 7αOHChol-induced expression of C5a receptor

**Fig 5:**
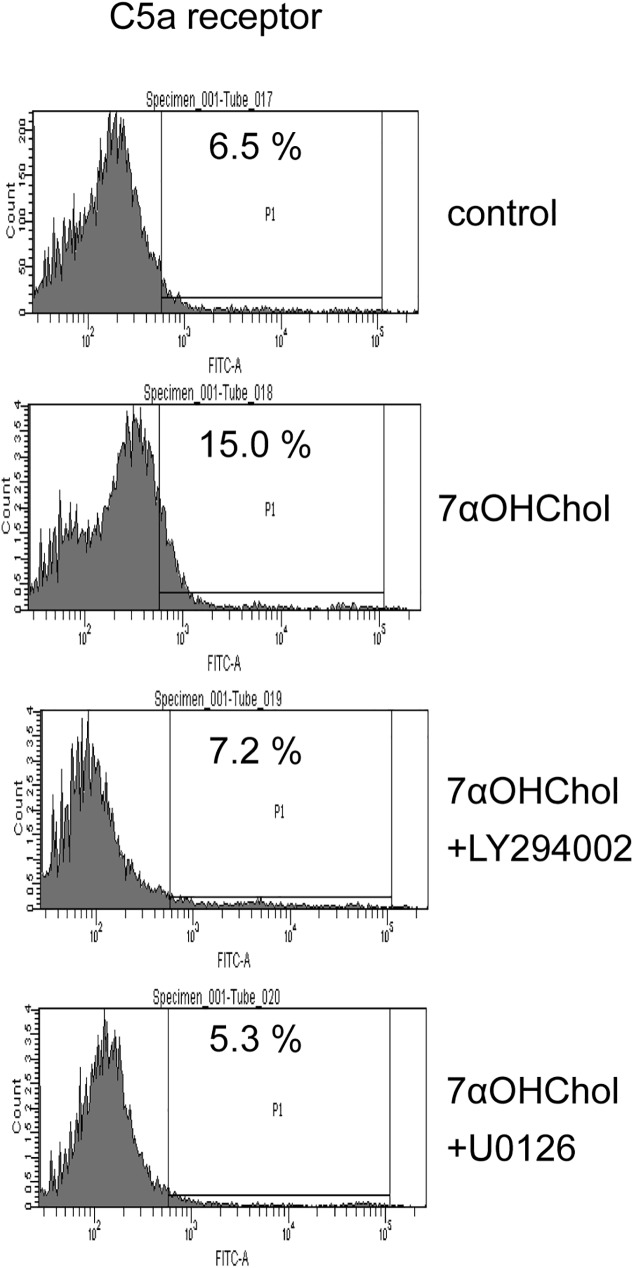
Effects of inhibitors of PI3K and MEK on expression of C5a receptor. THP-1 cells were treated with 7αOHChol for 48 h in the absence or presence of LY294002 or U0126 (10 μM each). The surface expression of C5a receptor was determined by flow cytometry.

Since inhibitors of the PI3K and the MEK attenuated 7αOHChol-induced transcription of the IL-8 gene in which C5a receptor is involved, we investigated whether inhibitors of the kinases affected expression of C5a receptor (Fig 5). The upregulated surface expression of C5a receptor by treatment with 7αOHChol was reduced to the basal level in the presence of LY294002 or U0126, meaning blockage of C5a receptor expression after inhibition of PI3K or MEK. Treatment with LY294002 or U0126 alone did not affect viability of THP-1 cells (S4 Fig), which indicated that inhibitory effects of the inhibitors were not due to cytotoxicity. Collectively, these results mean that expression of C5a receptor coincides with that of IL-8 via PI3K and MEK.

## Discussion

7-Oxygenated cholesterol derivatives detected in atherosclerotic lesions are biologically active molecules involved in atherogenic events. They cause cell death, but induce gene expression and cell differentiation at sub-cytotoxic concentrations. 7βOHChol and 7K induce apoptosis at high concentrations [15, 16]. 7αOHChol upregulates surface levels of Toll-like receptor 6 and induces production of CCL2, CCL3 and CCL4 and thereby promotes migration of monocytic cells and T lymphocytes expressing CCR5 [13, 17, 18]. 7K and 7αOHChol induces differentiation of monocytes into macrophages and mature dendritic cell phenotype, respectively [19, 20]. We have demonstrated elevated expression of IL-8 gene by 7αOHChol and 7K, which is consistent with previous reports that demonstrated expression of IL-8 after treatment of THP-1 cells with 7K [21, 22]. These findings suggest that individual 7-oxygenated cholesterol derivatives can contribute to progression of atherosclerosis via involvement in multiple, but distinct cellular and molecular events.

7αOHChol, 7K and 27OHChol elevate transcript levels of IL-8, but the elevation by the oxygenated cholesterol molecules occurs via different patterns. 7K induces and secretion of IL-8 as early as 6 h post-treatment [21, 22]. IL-8 gene transcription induced by 7αOHChol, which is detectable at 24 h post-treatment, is sustained through 72 h of post-treatment. 27OHChol-induced IL-8 gene transcription persists up to 48 h and disappears by 72 h of post-treatment [12]. The oxysterol molecules also show differences in their minimal concentrations to induce IL-8 transcription. Transcription of the IL-8 gene is induced in the presence of 0.5 μg/ml of 27OHChol whereas at least 2.5 μg/ml of 7αOHChol is needed to elevate transcription of the IL-8 gene. Taken together, these findings mean that 7K induces early expression of IL-8 and that 27OHChol is more effective at inducing expression of IL-8, in comparison with 7αOHChol, while 7αOHChol causes more sustained transcription of the IL-8 gene at high concentrations.

Atherosclerotic arteries express increased C5a receptor in contrast to normal coronary arteries, and apoE deficient mice express more C5a receptor mRNA in the aortas compared with wild-type mice [23, 24]. However, it is unknown whether 7-oxygenated cholesterol derivatives are involved in expression of C5a receptor. Results of this study suggest that 7-oxygenated cholesterol derivatives have differential effects on expression of C5a receptor because 7αOHChol alone induces expression of the receptor. Moreover, we have demonstrated involvement of C5a receptor in 7αOHChol-induced IL-8 expression, which is in line with the previous study by Hsu et al, which reported induction of IL-8 gene expression in mononuclear cells by treatment with a ligand for C5a receptor [25]. These findings suggest participation of C5a receptor in early inflammation in atherosclerotic lesions. We think that the inflammatory action of C5a receptor may explain the observation of reduced atherosclerosis by C5a inhibition in ApoE deficient mice [23].

Pharmacological inhibition of the PI3K/Akt or ERK pathway results in significantly reduced production of MMP-9, CCL2, and IL-23 and inhibition of the PI3K pathway leads to profoundly impaired migration of CCR5-positive Th1 lymphocytes enhanced by 7αOHChol [13, 17, 18]. Results of the current study further support the fact that both the PI3K/Akt and the ERK pathways are necessary for 7αOHChol to trigger or amplify inflammatory responses. It is well known that the two signaling pathways can cross-talk [26]; however, the current study did not determine whether the PI3K/Akt and the ERK pathways act in an independent or a cooperative manner. Future studies to elucidate the type of connection between the two pathways will provide better understanding of the molecular mechanisms underlying 7αOHChol-induced inflammation.

The 7-oxygenated cholesterol derivatives used this study are very similar in their structures. 7K has ketone, and 7αOHChol and 7βOHChol has α- and β-hydroxyl group at the 7 carbon position of cholesterol molecule, respectively [27]. The current study demonstrated expression of IL-8 and C5a receptor primarily by 7αOHChol in monocytes/macrophages. However, it is unknown how the 7-oxygenated derivatives induce different biochemical responses. It is possible that 7-oxygenated cholesterol derivatives may interact with distinct protein molecules. Further study is necessary to elucidate receptors and their signaling pathways utilized by each 7-oxygenated cholesterol derivative to understand molecular mechanisms underlying the differential effects of the oxygenated derivatives.

## Supporting information

S1 Fig Effects of 7-oxygenated cholesterol derivatives on cell viability. THP-1 cells were incubated for 48 h with or without the indicated 7-oxygenated cholesterol derivatives (5 μg/ml each). Cell viability was determined using a Vi-Cell cell counter (Beckman Coulter, Inc. Brea, CA). The viability of THP-1 cells cultured in medium alone was considered to be 100%. The viability of the cells treated with the oxygenated cholesterol derivatives was expressed as a percentage of the control value. Data are expressed as the means ± SD (n = 3 replicates for each group).
(TIF)

S2 Fig Effects of OxPAPC on IL-8 expression. THP-1 cells were incubated for 48 h with 7αOHChol (5 μg/ml) or for 9 h with peptidoglycan (PG) (1 μg/ml) in the absence or presence of OxPAPC (30 μM). Transcript levels of IL-8 were determined by real-time PCR. Data are expressed as the means ± SD (n = 3 replicates for each group). ns: non-significant; *** P < 0.001 vs. control; ### P < 0.001 vs. PG.
(TIF)

S3 Fig Effects of polymyxin B (PMB) on IL-8 expression. THP-1 cells were incubated for 48 h with 7αOHChol (5 μg/ml) or for 12 h with LPS (100 ng/ml) in the absence or presence of PMB (10 μg/ml). Transcript levels of IL-8 were determined by real-time PCR. Data are expressed as the means ± SD (n = 3 replicates for each group). ns: non-significant; *** P < 0.001 vs. control; ### P < 0.001 vs. LPS.
(TIF)

S4 Fig Effects of LY294002 and U0126 on cell viability. THP-1 cells were incubated for 48 h with or without the indicated inhibitors (10 μM each). Cell viability was determined using a Vi-Cell cell counter. The viability of THP-1 cells cultured with no inhibitor was considered to be 100%. The viability of the cells treated with each inhibitor was expressed as a percentage of the control value. Data are expressed as the means ± SD (n = 3 replicates for each group).
(TIF)
