# Supplementary figures and images for: 7α-Hydroxycholesterol induces monocyte/macrophage cell expression of interleukin-8 via C5a receptor
Source: PLoS One. 2017 Mar 21;12(3):e0173749. doi: 10.1371/journal.pone.0173749 (PMC5360241; doi:10.1371/journal.pone.0173749)

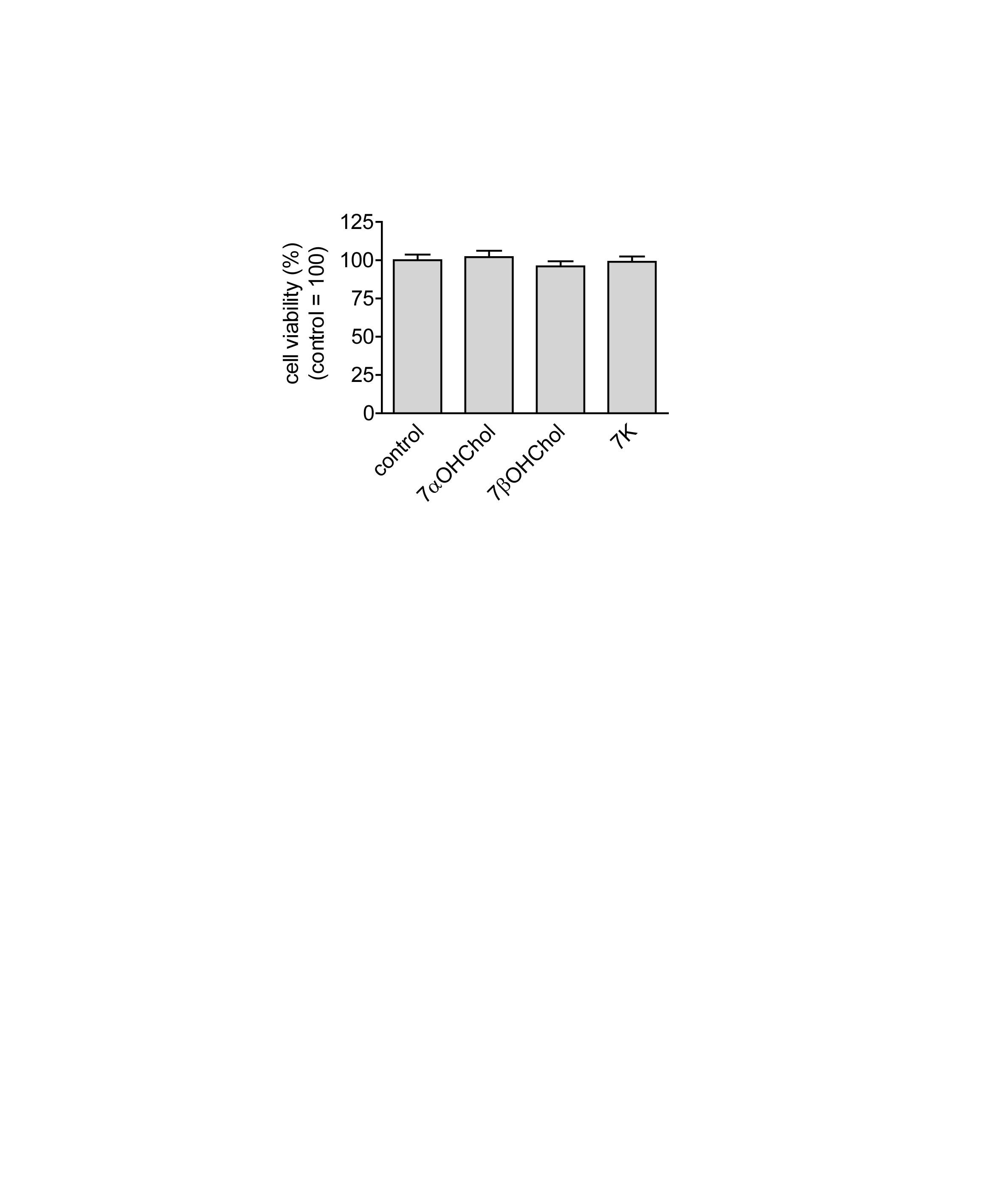

Supplement: S1 Fig — THP-1 cells were incubated for 48 h with or without the indicated 7-oxygenated cholesterol derivatives (5 μg/ml each). Cell viability was determined using a Vi-Cell cell counter (Beckman Coulter, Inc. Brea, CA). The viability of THP-1 cells cultured in medium alone was considered to be 100%. The viability of the cells treated with the oxygenated cholesterol derivatives was expressed as a percentage of the control value. Data are expressed as the means ± SD (n = 3 replicates for each group). (TIF) [file pone.0173749.s001.tif]

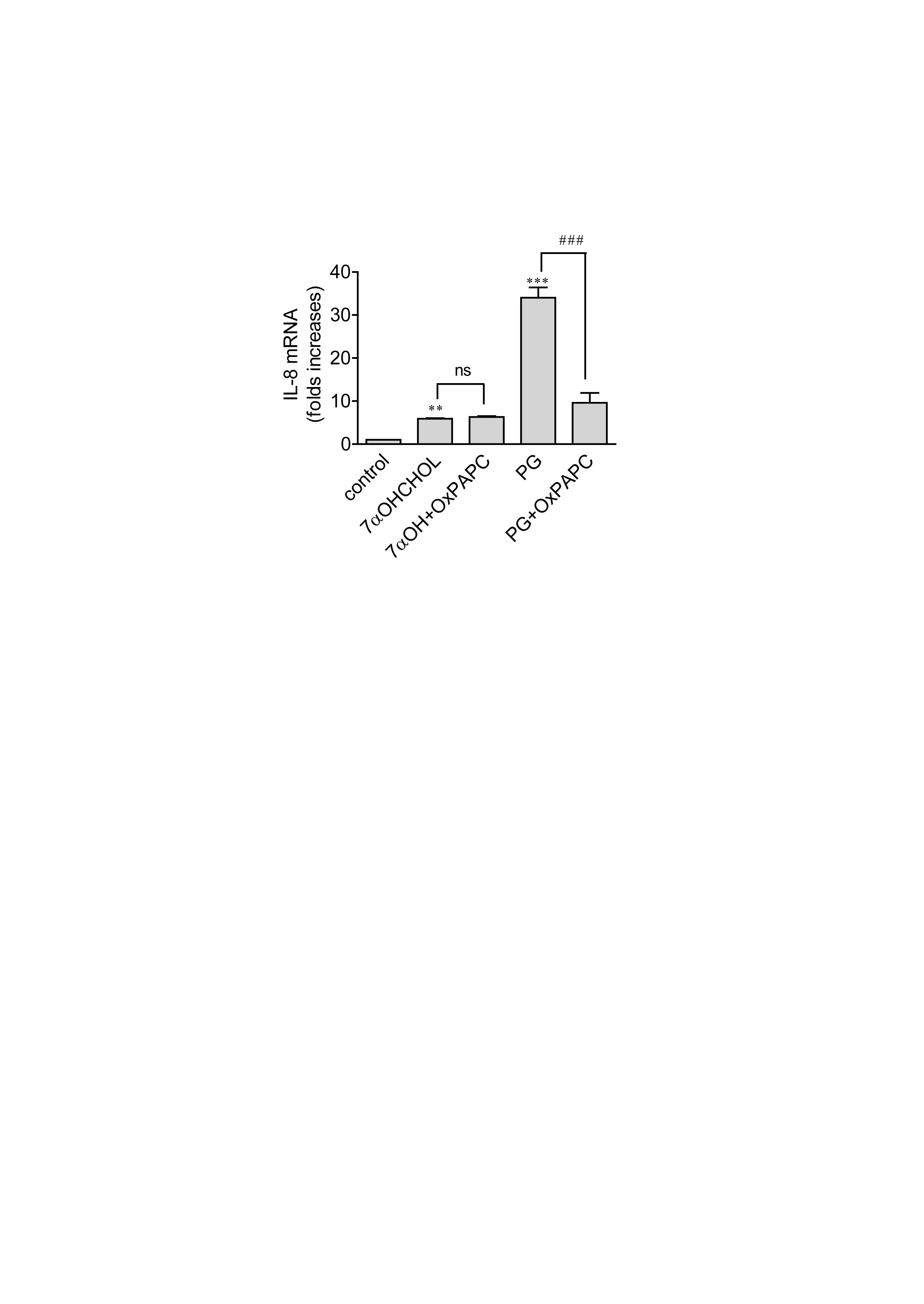

Supplement: S2 Fig — THP-1 cells were incubated for 48 h with 7αOHChol (5 μg/ml) or for 9 h with peptidoglycan (PG) (1 μg/ml) in the absence or presence of OxPAPC (30 μM). Transcript levels of IL-8 were determined by real-time PCR. Data are expressed as the means ± SD (n = 3 replicates for each group). ns: non-significant; *** P < 0.001 vs. control; ### P < 0.001 vs. PG. (TIF) [file pone.0173749.s002.tif]

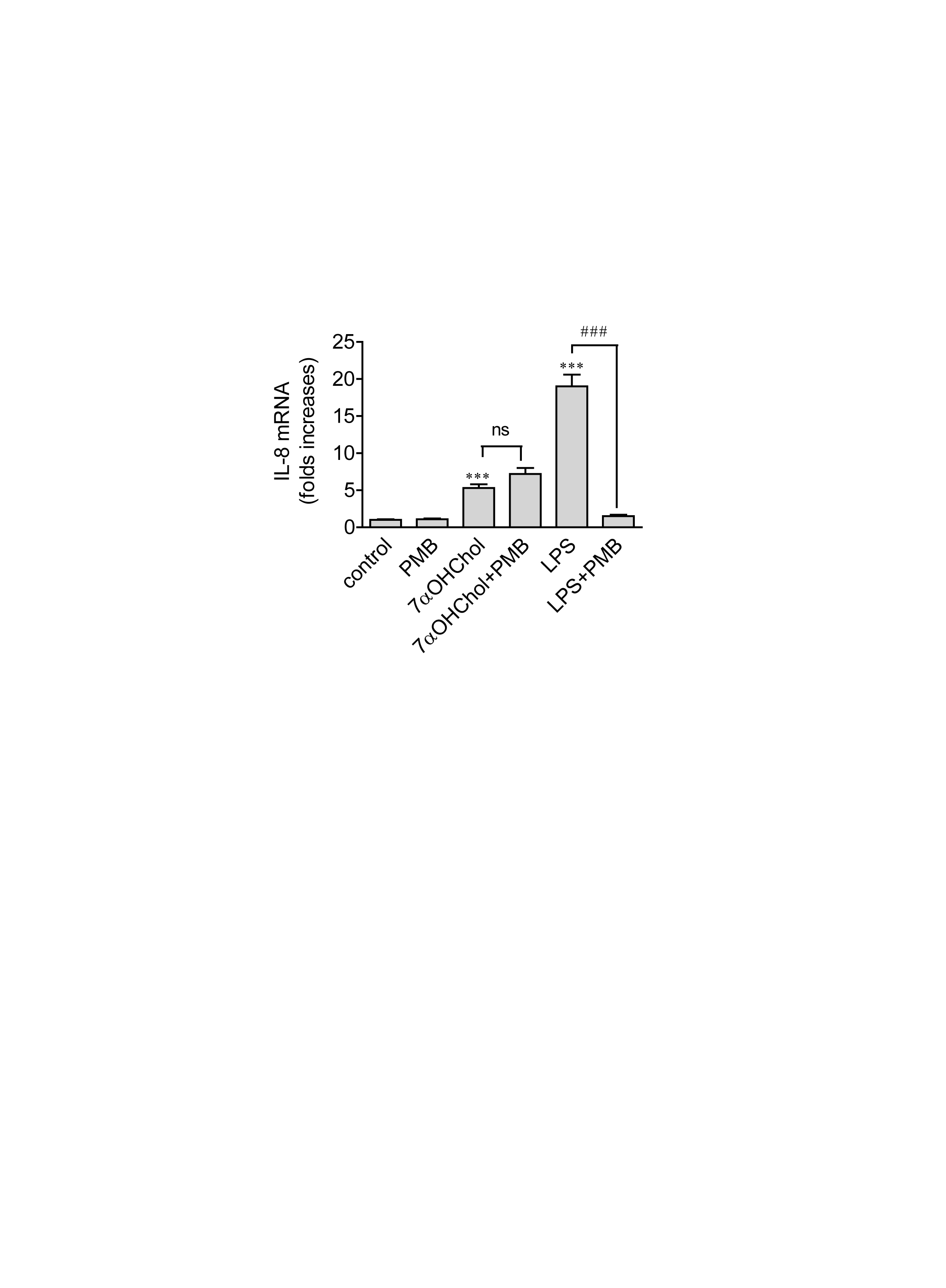

Supplement: S3 Fig — THP-1 cells were incubated for 48 h with 7αOHChol (5 μg/ml) or for 12 h with LPS (100 ng/ml) in the absence or presence of PMB (10 μg/ml). Transcript levels of IL-8 were determined by real-time PCR. Data are expressed as the means ± SD (n = 3 replicates for each group). ns: non-significant; *** P < 0.001 vs. control; ### P < 0.001 vs. LPS. (TIF) [file pone.0173749.s003.tif]

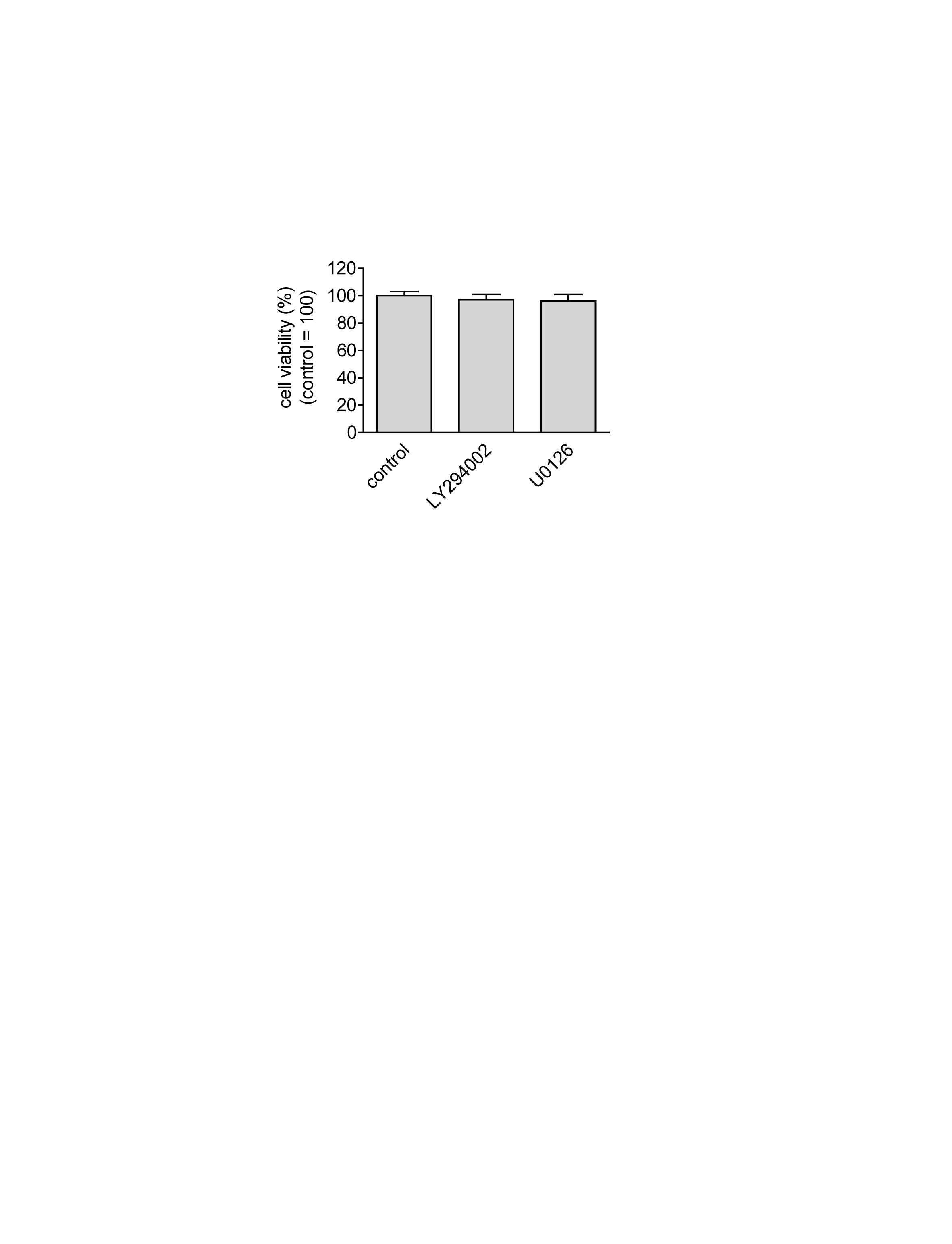

Supplement: S4 Fig — THP-1 cells were incubated for 48 h with or without the indicated inhibitors (10 μM each). Cell viability was determined using a Vi-Cell cell counter. The viability of THP-1 cells cultured with no inhibitor was considered to be 100%. The viability of the cells treated with each inhibitor was expressed as a percentage of the control value. Data are expressed as the means ± SD (n = 3 replicates for each group). (TIF) [file pone.0173749.s004.tif]
